# Tillage practices and straw-returning methods affect topsoil bacterial community and organic C under a rice-wheat cropping system in central China

**DOI:** 10.1038/srep33155

**Published:** 2016-09-09

**Authors:** Lijin Guo, Shixue Zheng, Cougui Cao, Chengfang Li

**Affiliations:** 1 MOA Key Laboratory of Crop Ecophysiology and Farming System in the Middle Reaches of the Yangtze River/College of Plant Science & Technology, Huazhong Agricultural University, Wuhan 430070, P.R. China; 2 State Key Laboratory of Agricultural Microbiology, College of Life Science and Technology, Huazhong Agricultural University, Wuhan 430070, P.R. China; 3 Hubei Collaborative Innovation Center for Grain Industry, Yangtze University, Jingzhou 434023, Hubei, P.R. China

## Abstract

The objective of this study was to investigate how the relationships between bacterial communities and organic C (SOC) in topsoil (0–5 cm) are affected by tillage practices [conventional intensive tillage (CT) or no-tillage (NT)] and straw-returning methods [crop straw returning (S) or removal (NS)] under a rice-wheat rotation in central China. Soil bacterial communities were determined by high-throughput sequencing technology. After two cycles of annual rice-wheat rotation, compared with CT treatments, NT treatments generally had significantly more bacterial genera and monounsaturated fatty acids/saturated fatty acids (MUFA/STFA), but a decreased gram-positive bacteria/gram-negative bacteria ratio (G^+^/G^−^). S treatments had significantly more bacterial genera and MUFA/STFA, but had decreased G^+^/G^−^ compared with NS treatments. Multivariate analysis revealed that *Gemmatimonas*, *Rudaea*, *Spingomonas*, *Pseudomonas*, *Dyella*, *Burkholderia*, *Clostridium*, *Pseudolabrys*, *Arcicella* and *Bacillus* were correlated with SOC, and cellulolytic bacteria (*Burkholderia, Pseudomonas, Clostridium, Rudaea* and *Bacillus*) and *Gemmationas* explained 55.3% and 12.4% of the variance in SOC, respectively. Structural equation modeling further indicated that tillage and residue managements affected SOC directly and indirectly through these cellulolytic bacteria and *Gemmationas*. Our results suggest that *Burkholderia, Pseudomonas, Clostridium, Rudaea*, *Bacillus* and *Gemmationas* help to regulate SOC sequestration in topsoil under tillage and residue systems.

Soil organic C (SOC) is the main source of energy for soil microorganisms^1^,^2^ and SOC content profoundly affects soil properties, including aggregate stability, soil moisture and nutrient cycling^3^. Thus, SOC plays an important role in maintaining long-term sustainability of agro-ecosystems and global biogeochemical cycles^4^. SOC is regulated by many factors, such as tillage practices^1^, residue management^5^, soil aggregate sizes^6^, and microbial functional diversity^2^. Optimizing agricultural management can reduce SOC loss, or even increase the content of SOC^7^. Intensive and continuous soil tilling has been practiced for thousands of years in China^5^. Frequent soil disturbance by intensive conventional tillage (CT) reduces soil aggregate sizes, thereby accelerating SOC oxidation^8^ and decreasing SOC content. Moreover, crop residue burning or removing, a common farming practice, reduces the amount of organic substances retained in the soil and the water storage capacity of the entire soil^9^, and decreases soil microbial biomass and functional diversity^2^. In contrast, no-tillage (NT) and straw returning (S) may enhance SOC content in agricultural ecosystems and facilitate sustainable agricultural production^5^,^7^.

The abundance, diversity and composition of soil microbial communities and their interactions with environment factors have great impacts on SOC dynamics^6^,^10^,^11^,^12^. In agricultural ecosystems, bacteria and fungi are the main drivers of soil processes including nutrient cycling and the decomposition of soil organic matter, such as crop residues^13^,^14^,^15^. Tillage practices and straw-returning methods affect the activity and community structure of soil microorganisms by changing the habitat characteristics for soil microorganisms such as soil porosity, soil moisture and the substrates for soil microorganisms^16^, thus affecting SOC dynamics in soil ecosystem. Although many studies have shown that soil fungi are a major factor influencing soil carbon content, these studies were focused on upland ecosystems, such as forest ecosystems^17^,^18^. In rice-wheat system, the field is long-term flooded and is mostly under anaerobic conditions during rice growing period^19^, which thus inhibits the growth of fungi and reduces the contributions of fungi to SOC. Some studies reported that bacteria are dominant in rice-wheat system^7^, which may be due to their ability to break down labile carbon sources more efficiently than other microorganisms, such as fungi^20^, thus contributing to the increase of SOC concentration through the binding of fresh and labile pools of organic matter with microaggregates to form macroaggregates^21^. Therefore, bacteria may have greater contributions to SOC concentration than fungi in the rice-wheat system.

Most of previous studies focused on the effects of tillage practices and straw-returning methods on soil bacterial abundance, and consistently showed that both NT and S practices can increase soil bacterial abundance^7^,^22^. For example, Guo *et al*.^7^ reported higher soil bacterial abundance under NT and S treatments than under CT and NS treatments, respectively, possibly because both NT and S practices provide more favorable environmental conditions for soil microorganisms^23^. Zhang *et al*.^22^ also reported that NT significantly increased bacterial biomass compared with CT. However, these studies ignored the relationships between soil bacterial communities and SOC. Zhang *et al*.^6^ investigated the contribution of soil biota (including bacteria) to C sequestration under different tillage practices, and showed that microbial communities controlled C storage both directly and indirectly through MBC and soil bacteria contributed to C sequestration both in <1 mm and >1 mm soil aggregates. Guo *et al*.^1^ reported the relationships between microbial metabolic characteristics and SOC within aggregates under different tillage practices and straw-returning methods, and indicated that the increased SOC in aggregates in the topsoil under NT and S practices was possibly due to the improvement of microbial metabolic activities. Nevertheless, the mechanism by which different functional genera of soil bacteria are linked to SOC sequestration under different tillage practices and their relative contributions remain unknown. Therefore, further investigation is needed to understand the relative contributions of soil bacterial communities to SOC and how these relationships may vary under different tillage practices.

Rice-wheat cropping system, which occupies a total area of 4.5 million ha in China, possesses important functions in food security in the world^24^. However, the sustainability of rice-wheat cropping system is negatively affected by issues such as soil degradation, air pollution^25^ and the long-term use of conventional management practices, such as crop residue removing or burning, or intensive soil tilling^7^. Rice-wheat cropping system is the most important cropping system in central China^26^, occupying about 20% of the total sown area in central China and accounting for about 22% of the national grain yield for these two crops in 2011^27^. The effects of tillage practices and straw-returning methods on soil physical-chemical properties, soil nutrient and crop yield under rice-wheat cropping system in this region have been well elucidated^23^,^26^. However, little attention has been paid to the relationships between bacterial communities and SOC under different tillage practices and straw-returning methods in this region. The objective of this study was to assess the effects of tillage practices (i.e. NT and CT) and straw returning methods (i.e. crop residue removal (S) and returning (NS)) on topsoil bacterial communities and their relationships with SOC under rice-wheat cropping system in central China. We hypothesized that (1) NT and S practices can improve soil bacterial abundance mainly due to the improvement of soil nutrition condition in the topsoil layer, and (2) the bacterial communities can have positive effects on SOC under NT and S practices. Structural equation modeling (SEM) was used to detect the potential associations among tillage systems/straw systems, bacterial communities and SOC.

## Results

### Phospholipid fatty acid (PLFA) analysis

After a 2-year cropping cycle, NT and S practices significantly affected the composition of soil microbial communities in the 0–5 cm soil layer. Compared with CT and NS treatments, NT and S treatments had significantly higher total PLFAs, bacterial PLFA, gram-positive bacterial PLFA, gram-negative bacterial PLFA and MUFA/STFA, and significantly lower G^+^/G^−^ (Table 1 and Supplementary information).

### Relationships between PLFA and SOC fractions

**Figure 1:**
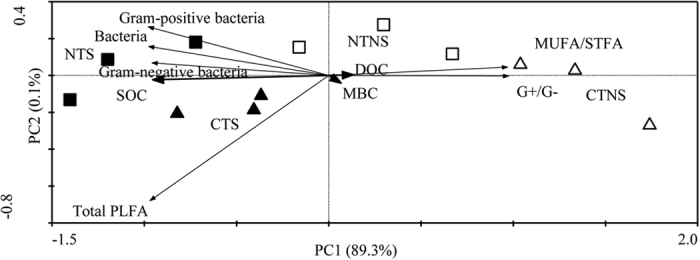
Redundancy analysis of soil microbial communities and SOC fractions under different treatments. SOC, soil organic C; MBC, microbial biomass C; DOC, dissolved organic C; MUFA/STFA, monounsaturated fatty acids/saturated fatty acids; CTNS (▵), conventional intensive tillage with straw removal, CTS (▴), conventional intensive tillage with straw returning; NTNS (□), no-tillage with straw removal; NTS (■), no-tillage with straw returning.

Redundancy analysis showed that the coordinates from the first two ordination axes explained 89.4% (the first axis 89.3% and the second 0.1%) of the variances (Fig. 1). A Monte Carlo permutation test showed that SOC fractions (including SOC) were significantly correlated with the differences in the composition of the soil microbial community (*P* < 0.05). Moreover, SOC was the most closely related to G^+^/G^−^ and MUFA/STFA. Overall, a clear separation was found between treatments (Fig. 1).

### Soil bacterial community structure

**Figure 2:**
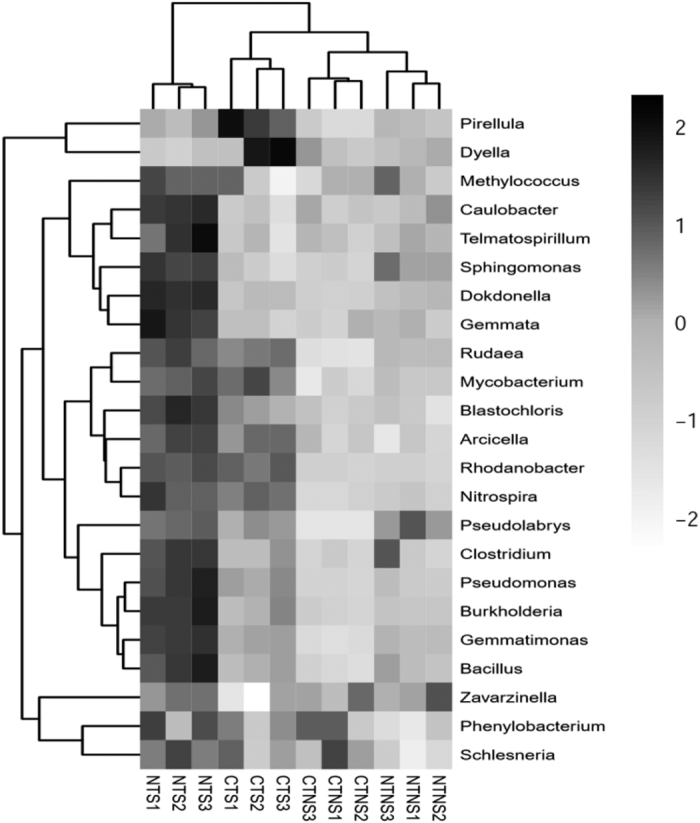
Relative abundance (n = 3) of top 23 OTUs of bacteria genera under different treatments revealed by pyrosequencing. CTNS, conventional intensive tillage with straw removal; CTS, conventional intensive tillage with straw returning; NTNS, no-tillage with straw removal; tillage; NTS, no-tillage with straw returning.

In general, NT treatments had significantly greater bacterial abundance compared with CT treatments, and S treatments had significantly higher bacterial abundance of 11 main soil genera compared with NS treatments (Fig. 2, Table 2 and Supplementary information). Compared with CT treatments, NT treatments had significantly greater abundance of *Gemmatimonas*, *Rudaea*, *Sphingomonas*, *Caulobacter*, *Dokdonella*, *Telmatospirillum*, *Pseudomonas*, *Burkholderia*, *Pseudolabrys*, *Blastochloris*, and *Bacillus*. Compared with NS treatments, S treatments had significantly higher abundance of *Gemmatimonas*, *Rudaea*, *Sphingomonas*, *Dokdonella*, *Rhodanobacter*, *Mycobacterium*, *Nitrospira*, *Gemmata*, *Schlesneria*, *Pseudomonas*, *Pirellula*, *Burkholderia*, *Clostridium*, *Pseudolabrys*, *Blastochloris*, *Arcicella* and *Bacillus*.

### Relationship between soil bacterial communities and SOC fractions

**Figure 3:**
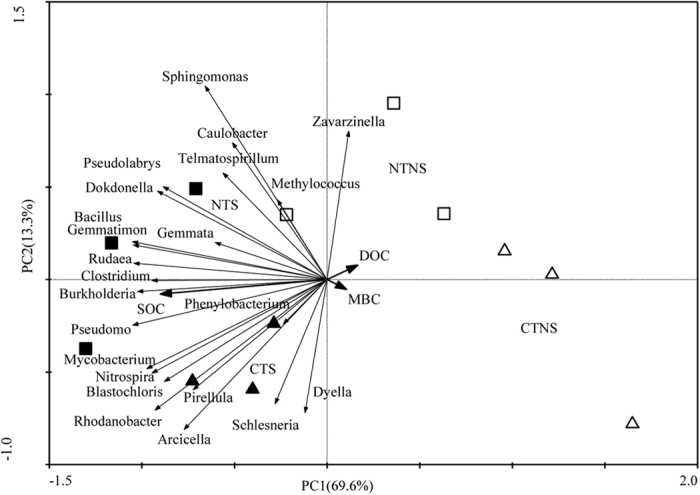
Redundancy analysis of soil bacterial communities and SOC fractions under different treatments. CTNS (▵), conventional intensive tillage with straw removal; CTS (▴), conventional intensive tillage with straw return; NTNS (◽), no-tillage with straw removal; NTS (◾), no-tillage with straw returning; SOC, soil organic C; MBC, microbial biomass C; DOC, dissolved organic C.

Redundancy analysis showed that the coordinates from the first two ordination axes explained 82.9% (the first axis 69.6% and the second 13.3%) of the variances (Fig. 3). A Monte Carlo permutation test showed that all SOC fractions (including SOC) were significantly correlated with the differences in the abundance of bacterial communities (*P* < 0.05). Moreover, SOC was positively correlated with the abundance of *Burkholderia*, *Pseudomonas*, *Clostridium*, *Rudaea*, *Gemmatimonas* and *Bacillus*. Clear separations could be seen between treatments in the redundancy analysis (Fig. 3).

### Stepwise regression analysis

*Gemmatimonas*, *Rudaea*, *Spingomonas*, *Pseudomonas*, *Dyella*, *Burkholderia*, *Clostridium*, *Pseudolabrys*, *Arcicella* and *Bacillus* were significantly related to SOC (Table 3).

### Relative importance analysis

**Figure 4:**
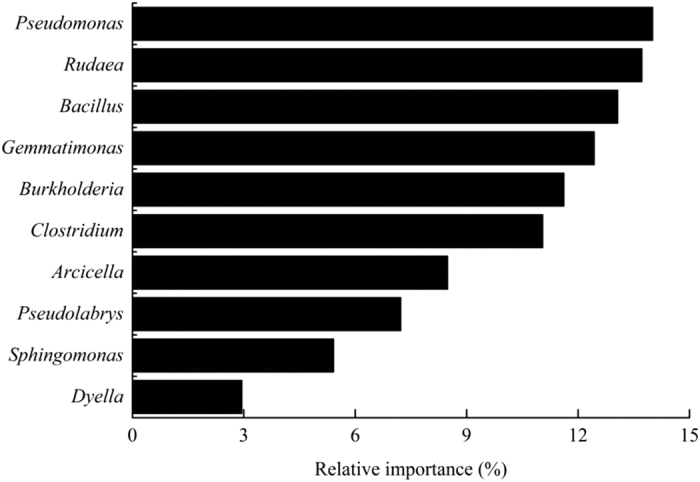
Relative importance analysis of the soil bacterial genera to SOC by R (relative importance package) based on the results of stepwise regression analysis.

All bacterial genera could explain 86.6% of the variances in SOC (Fig. 4). *Pseudomonas*, *Rudaea*, *Bacillus*, *Gemmatimonas*, *Burkholderia* and *Clostridium* were the main influencing factors to SOC, which together explained 75.6% of the variances.

### Links between soil bacterial communities and SOC

**Figure 5:**
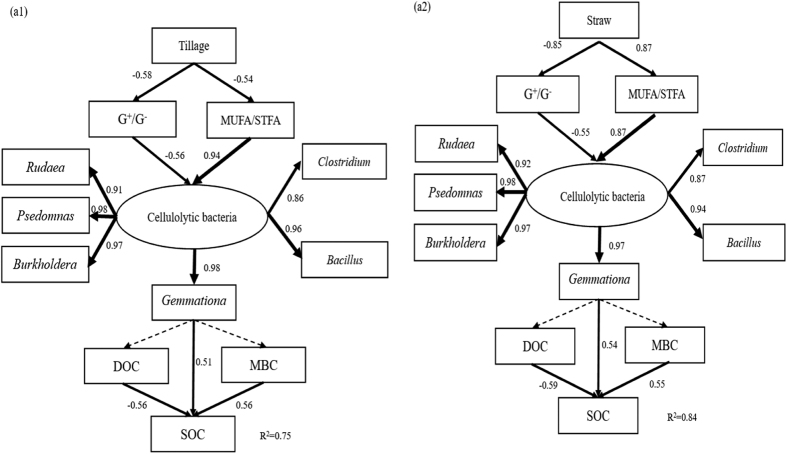
Structural equation model relating tillage systems, straw systems and bacteria communities to SOC (a1, χ^2^ = 124.867, df = 51, p = 0.525, CFI = 0.952, GFI = 0.923, RMSEA = 0.010; a2, χ^2^ = 114.847, df = 51, p = 0.565, CFI = 0.962, GFI = 0.915, RMSEA = 0.001). Rectangles represent observed variables. Arrow thickness represents the magnitude of the path coefficient. Values associated with solid arrows represent the path coefficients. Solid and dashed arrows indicate significance (*P* < 0.05) and non-significance (*P* > 0.05), respectively. Straw, straw systems; DOC, dissolved organic C; MBC, microbial biomass C; SOC, soil organic C.

SEM revealed that the predictors explained 75.0–84.0% of the variances in SOC (Fig. 5). In Fig. 5a1,a2, tillage and straw systems had different levels of MUFA/STFA and G^+^/G^−^, and thus likely affected SOC directly and indirectly through the presence of 5 kinds of cellulolytic bacteria (*Pseudomonas*, *Rudaea*, *Bacillus*, *Burkholderia* and *Clostridium*) and *Gemmatimonas*.

## Discussion

This study investigated the effects of tillage practices and straw-returning methods on soil bacterial communities and their relation to SOC after a 2-year rice-wheat cropping cycle in central China. The results supported our hypotheses that NT and S practices increase the abundance of bacterial genera in the topsoil bacterial communities, and that the composition of the bacterial community is correlated with SOC.

### Effects of tillage practices and straw returning methods on soil bacterial community

NT and S practices generally increased the abundance, activity and diversity of soil microbial communities in the topsoil layer^2^,^28^,^29^, probably because NT minimizes soil disturbance and S contributes to greater accumulation of crop residues on the soil surface^8^, thus improving soil nutrition condition for soil microbial communities^7^. Soil nutrition condition can be indicated by G^+^/G^−^ ratio^30^. Lower G^+^/G^−^ ratio under NT treatments compared with under CT treatments (Table 1) suggests that nutrients are rich in the topsoil layer under NT treatments, which demonstrates the improvement of soil environment for microorganisms under NT^7^. Guo *et al*.^7^ and Wang *et al*.^31^ also reported that NT could significantly increase soil N, organic C and SOC fractions compared with CT, whereas CT could negatively affect soil microbial biomass and SOC. Moreover, a greater MUFA/STFA ratio in NT treatments compared with in CT treatments (Table 1) suggests that NT treatments may improve soil gas permeability as suggested by Bossio *et al*.^32^, because of the accumulation of crop residues on the soil surface under NT^8^.

Straw returning, as an input of organic residues to improve soil nutrition condition, can increase soil surface residue C and SOC^7^ and provide energy sources for soil microbes, thus enhancing soil microbial biomass^2^,^7^. Residue amendment improves soil moisture and temperature and promotes soil aggregation, thus boosting microbial growth^20^, which is supported by the result of lower G^+^/G^−^ under S treatments than under NS treatments (Table 1). However, in the study of the impacts of residue management on soil properties and soil microbial community structure, Wang *et al*.^33^ did not find significant differences in bacterial abundance between S and NS treatments. In addition, S treatments had a higher MUFA/STFA ratio compared with NS treatments (Table 1). This result indicates that soils under S treatments may have greater gas permeability^32^, possibly because straw returning decreases the sensitivity to surface sealing^34^ and increases the porosity of the top soil layer^35^. Good soil gas permeability and enrichment of organic matter in soil surface under NT and S practices^7^,^8^ also promote the decomposition of exogenous crop straw, thus improving soil nutrition condition. Therefore, NT and S practices improve soil nutrition, leading to the increase of soil bacterial abundance in the topsoil layer.

### Relationships between soil bacterial communities and SOC

Our results showed that there were seven predominant bacterial genera (*Gemmatimonas*, *Rudaea*, *Caulobacter*, *Sphingomonas*, *Dokdonella*, *Rhodanobacter* and *Mycobacterium*) in the 0–5 cm soil layer, which accounted for 67.7% of total bacterial abundance (Fig. 2). Multiple analysis results showed that soil bacterial communities were closely related to SOC, and *Pseudomonas*, *Rudaea*, *Bacillus*, *Gemmatimonas*, *Burkholderia* and *Clostridium* greatly contributed to SOC, together explaining 75.6% of the variances in SOC. *Pseudomonas*, *Rudaea*, *Bacillus*, *Clostridium*, *Burkholderia* and *Dyella* belong to cellulolytic bacteria^32^, and together explained 66.3% of the variances in SOC, suggesting that SOC is mainly regulated by these six cellulolytic bacteria. Cellulose is unavailable to most soil microorganisms because the crystallinity of cellulose is extremely recalcitrant for enzymatic degradation^36^. Some studies have suggested that cellulolytic bacteria help to regulate the C cycle^37^ because they play an important role in the decomposition of plant residues in the soil ecosystem^36^.

In this study, most of the cellulolytic bacteria screened by stepwise regression analysis are aerobic microorganisms (Table 3), which can be attributed to the high permeability in the 0–5 cm soil layer^35^. Generally, cellulose is mainly degraded in aerobic environments, while up to 5–10% of cellulose is degraded by physiologically diverse bacteria under anaerobic conditions^37^. Many studies have indicated that *Clostridium*, one of important cellulolytic anaerobic bacterial genera^38^, is highly efficient in degrading cellulose^36^,^39^ because it excretes several kinds of enzymes including cellulase and hemicellulase^40^. In the present study, NT and S treatments had significantly greater *Clostridium* abundance (Fig. 2). Multiple analyses suggested that *Clostridium* may play important roles in SOC (Figs 1, 2, 3 and 4). *Clostridium* is negatively affected by greater oxygen availability in the soil and soil disturbance^8^. Hence, its high abundance under NT and S treatments was not unexpected as it is likely that the higher residue mulching under S practice and/or less soil disturbance under NT^1^,^7^ create anaerobic zones in the surface soil.

*Gemmatimonas* (22.6%, 245 OTUs) is the most abundant bacterial genera in this study (Fig. 2), and explained 12.4% of the variances in SOC (Fig. 4). The SEM also showed the key function of *Gemmatimonas* to SOC sequestration under NT and S practices in this study (Fig. 5). The reason may be that *Gemmatimonas* can use the metabolic products as sole C sources^41^, such as acetate and propionate^42^,^43^,^44^, but most of other bacterial genera, such as *Bellilinea*^45^ and *Sphingomonas*^46^ (Fig. 2), cannot or can only weakly use the metabolic products of cellulose. Thus, it is likely that *Gemmatimonas* has greater ability to use available C sources compared with other soil microorganisms. The SEM further showed that *Gemmatimonas* plays an important role in SOC dynamics (Fig. 5), which can be attributed to the fact that *Gemmatimonas* can reduce the metabolic products of cellulose^41^ and thus indirectly promotes the degrading process of cellulose.

Tillage practices and straw returning methods affect the activity and structure of soil microorganisms by changing the habitat characteristics for soil microorganisms such as soil gas permeability and the substrates for soil microorganisms^16^,^33^, thus affecting SOC. Both NT and S practices promote the accumulation of straw on the soil surface, in which the major component is cellulose^47^, thus improving soil physical conditions^21^ and also providing C sources (specifically cellulose) for cellulolytic bacteria^36^. Therefore, NT and S practices promoted the growth of cellulolytic bacteria (Fig. 2), thus increasing the decomposition of cellulose and subsequently the SOC (Figs 2, 3, 4 and 5 and Table 3). Decomposition of exogenous crop straw provides C sources for other soil microorganisms, and therefore increases soil microbial biomass^48^, which contributes to the developing and increasing of soil organic matter^2^,^6^. However, exogenous organic matter from broken down cellulose promotes C sequestration in soil aggregates, especially in >250 μm aggregates because the broken down exogenous organic matter could be bound to the walls of the mineral particles that surround them^2^,^21^. Yin *et al*.^49^ also reported that bacteria play critical roles in the production of soil aggregates and the conversion of plant residue to soil organic matter. The results of this study suggest that tillage changes the habitats for *Pseudomonas, Rudaea, Bacillus, Burkholderia, Della* and *Clostridium*, and then changes the decomposition process of residue, thus affecting SOC in the 0–5 cm soil layer.

This study indicates that after two cycles of rice-wheat rotation, NT and S practices promote SOC in the 0–5 cm soil layer presumably by increasing the abundance of bacterial genera. Redundancy analysis showed a close relationship between SOC levels and the abundance of specific bacterial genera in the soil community. Stepwise regression analysis and relative influence analysis indicated that *Gemmatimonas*, *Rudaea*, *Spingomonas*, *Pseudomonas*, *Dyella*, *Burkholderia*, *Clostridium*, *Pseudolabrys*, *Arcicella* and *Bacillus* are positively correlated with SOC. SEM results further suggested that NT and S practices specifically increase the abundance of 5 kinds of cellulolytic bacteria (*Burkholderia, Pseudomonas, Clostridium, Rudaea*, and *Bacillus*) and *Gemmatimonas* in the upper soil layer, likely promoting SOC levels. However, the mediation of bacterial communities on SOC under long-term NT and S practices in the rice-wheat cropping system should be further discussed. Long-term (5+ years) NT and S practices may change SOC in the whole plough layer (0–20 cm); however, the ability of bacterial communities to regulate these effects remains unclear. Therefore, further studies should be conducted to reveal the mechanism of the effects of long-term NT and S practices on soil bacterial communities and their contributions to SOC in the whole plow layer.

## Methods

### Experimental site

The study site was located at an experimental farm of Huazhong Agricultural University Research (29°51′N, 115°33′E) in the town of Huaqiao Town, Wuxue City, Hubei Province, China, which has been described by Guo *et al*.^2^. The soil is a silty clay loam classified by the Food and Agriculture Organization (FAO) as a Gleysol^2^. The experimental soil (0–20 cm depth) has a pH of 4.79, an organic C content of 16.89 g kg^−1^, a total nitrogen (N) content of 2.20 g kg^−1^, a total phosphorus (P) content of 0.45 g kg^−1^, and a bulk density of 1.21 g cm^−3^. The cropping regime was dominated by two crops: summer rice (HHZ, *Oryza sativa* L.) and winter wheat (ZM9023, *Triticum aestivum* L.).

### Experimental design

The detailed experimental design was described by Guo *et al*.^2^. In brief, field treatments followed a split-plot design of a randomized complete block with tillage practices (conventional intensive tillage, CT; no tillage, NT) as the main plots and straw returning methods [crop straw removal (NS) and crop straw return (S)] as the subplots. The experiment involved four treatments: CTNS, CTS, NTNS and NTS, with each replicated for three times. For CTNS and NTNS treatments, crop residues were removed and not returned to the field. For CTS and NTS treatments, residues were chopped into pieces 5–7 cm in length and returned to the field. The chopped straw was mulched in NT soil and tilled into CT soil. For CT treatments, the soil was moldboard ploughed twice to a 20 cm depth before throwing of rice seedlings and once before sowing of wheat. The soil was not disturbed for NT treatments. Commercial compound fertilizer (15% N, 15% P_2_O_5_, and 15% K_2_O), urea (46% N), single superphosphate (12% P_2_O_5_), and potassium chloride (60% K_2_O) were used to provide 180 kg N ha^−1^, 90 kg P_2_O_5_ ha^−1^, and 180 kg K_2_O ha^−1^ during the rice-growing seasons, and 144 kg N ha^−1^, 72 kg P_2_O_5_ ha^−1^, and 144 kg K_2_O ha^−1^ during the wheat-growing season. P and K fertilizers were only applied as basal fertilizers, and N fertilizers were used with 50%, 20%, 12%, and 18% at the seedling, tillering, jointing, and earring stages of rice-growing seasons, and with 50%, 30%, and 20% at the seedling, tillering, and boosting stages of wheat-growing seasons, respectively. The plots were irrigated to a depth of 8 cm whenever the water depth above soil surface decreased for 1–2 cm during the rice growing season, and were drained in the tillering and maturing stages. We did not irrigate during the wheat-growing season.

### Soil sampling

Soil samples were collected from the topsoil (0–5 cm depth) using a soil sampler (7 cm diameter) immediately after wheat harvest in June 2013 at eight random points in each plot. After sampling, visible plant residues and stones were removed, and large soil clods were gently broken by hand. Soils were sieved through a 5 mm screen for uniformity, and stored at −20 °C, and all determinations were finished within two weeks.

The SOC and its fractions (microbial biomass C (MBC) and dissolved organic C (DOC)) in the 0–5 cm soil layer were reported previously by Guo *et al*.^2^.

### Phospholipid fatty acid (PLFA) analysis

PLFA analysis was conducted to measure the composition of soil microbial communities according to the methods of Blair *et al*.^50^ and Bossio *et al*.^32^ and detailed measurements were performed as described by Guo *et al*.^7^. Briefly, lipids were extracted in a single-phase chloroform-methanol-citrate (1:2:0.8) buffer system. Polar lipids were separated from neutral lipids and glycolipids on solid phase extraction columns (Supelco Inc, Bellefonte, PA, USA) by eluting with CHCl_3_, acetone, and methanol. The phosholipid fractions were saponified and methylated to fatty acid methyl esters (FAME). Nonadecanoic acid methyl ester was used as internal standard and was added to calculate the absolute amounts of FAMEs before measurements. PLFAs were analyzed as FAMEs on a gas chromatograph/mass spectrometry system (6890–5973N series GC/MS Agilent Technologies, Palo Alto, CA, USA).

### DNA extraction, PCR amplification, 16S rDNA gene amplification and 454 pyrosequencing

High-throughput sequencing technology, a common method for identifying bacterial communities in various habitats and environmental samples, was used to identify bacteria in the soil samples^51^. Total soil DNA was extracted using a FastDNA^®^ Kit for soil (MP Biomedicals, Santa Ana, CA, USA) according to the manufacturer’s instructions. The concentration and quality (A260/A280) of the soil DNA were measured by a NanoDrop ND-2000 spectrophotometer (NanoDrop, Wilmington, DE, USA). The DNA of each sample was diluted 50-fold and stored at −20 °C, and all determinations were completed within two weeks.

An aliquot (10 ng) of purified DNA from each sample (one biological replicate) was used as template for amplification. The primer 357F (5′-CCTACGGGAGGCAGCAG-3′), which was modified with the addition of the 454 FLX-titanium adaptor “B” sequence (5′-CCTATCCCCTGTGTGCCTTGGCAGTCTCAG-3′), was used to amplify the V3, V4 and V5 hypervariable regions of the bacterial 16S rDNA^52^, and 926R: 5′-CCGTCAATTCMTTTRAGT-3′ was modified with the addition of unique 6–8 nucleotide barcode sequences and the 454 FLX-titanium adaptor “A” sequence (5′-CCATCTCATCCCTGCGTGTCTCCGACTCAG-3′)^52^. Each sample was amplified in triplicate in a 25 μl reaction and the program was as follows: initial denaturation at 95 °C for 4 min, followed by 25 cycles of denaturation (94 °C for 30 s), annealing (55 °C for 45 s), extension (72 °C for 1 min), and a final elongation step for 8 min at 72 °C. PCR Purification Kit (Axygen, Union City, CA, USA) was used to purify the PCR products. The amplicons of each sample were then pooled in equimolar concentrations into a single tube prior to 454 pyrosequencing. Pyrosequencing was performed on a 454 GS-FLX Titanium System (Roche, Basel, Switzerland) by Shanghai Personal Biotechnology Co., Ltd. Quality filtering of data was conducted following Fierer *et al*.^53^ using the Quantitative Insights into Microbial Ecology (QIIME) pipeline (http://qiime.sourceforge.net)^54^. In brief, sequences with an average quality score of less than 25, sequences with lengths less than 200 nt or greater than 1,000 nt, with ambiguous bases greater than 1, with homopolymer lengths greater than 6, or with maximum primer mismatches greater than 0 were removed from the dataset. And chimeric sequences were removed using the uchime algorithm in mothur (Version 1.21.2, http://www/mothur.org/)^55^. Sequences were clustered into operational taxonomic units (OTUs) using the QIIME implementation of cd-hit with a threshold of 97% pairwise identity. The longest sequences were extracted and taken as representatives for taxonomic identification by BLAST searches against the non-redundant GenBank sequence database. In order to study the function of soil bacterial communities in the SOC dynamics, the relative abundance of soil bacterial genera (OTUs) was used for multiple analysis in this study.

### Statistical analyses

General linear model analysis of variance with SAS 9.0 designed for split plot with tillage practice and straw returning methods as fixed factors and replicates as random factors was conducted to test the main effects and interactions of tillage and straw returning. The least significant difference (LSD) test was used determine the significance of the effects of tillage, straw returning or their interactions. Only the means statistically different at *P* ≤ 0.05 were considered. Detrended correspondence analysis performed by CANOCO software showed that the data of characteristics of soil microbial communities and bacterial abundance were fitted with the linear model. Thus, redundancy analysis was performed using CANOCO software to explain the relationships between SOC fractions and bacterial communities. Monte Carlo permutation test performed by Cannoco 4.5 was used to assess the statistical significance of explanatory variables. Stepwise regression analysis (SAS 9.0) was performed to determine the relationships between SOC and bacterial genera. The contributions of bacterial genera to SOC were estimated by the relative importance analysis, using the “relaimpo” package in R^56^. Structural equation modeling (SEM), a multivariate statistical method that enables hypothesis testing of complex path-relation networks^57^, was used to evaluate whether bacterial communities mediate the change of SOC in response to the conversion of CT to NT, or NS to S. We constructed an *a priori* model according to a literature review and our knowledge of how these predicators are related. The initial model comprised eight predictors: tillage systems (Tillage), straw systems (Straw), monounsaturated fatty acids/saturated fatty acids (MUFA/STFA), gram-positive bacteria/gram-negative bacteria (G^+^/G^−^), dissolved organic carbon (DOC), microbial biomass carbon (MBC), soil organic carbon (SOC), and key genera of the soil bacterial communities (*Pseudomonas, Rudaea, Bacillus, Burkholderia*, *Clostridium*, and *Gemmatimonas* picked by stepwise regression analyses), which greatly contributed to the SOC. A ‘robust’ maximum likelihood estimation procedure of AMOS 7.0 software was conducted for the analysis. χ^2^-test, comparative fit index (CFI), goodness-of-fit (GFI) and root square mean error of approximation (RMSEA) were performed to evaluate model fit.

## Additional Information

**How to cite this article**: Guo, L. *et al*. Tillage practices and straw-returning methods affect topsoil bacterial community and organic C under a rice-wheat cropping system in central China. *Sci. Rep.* **6**, 33155; doi: 10.1038/srep33155 (2016).

## Supplementary Material

Supplementary Information

## Figures and Tables

**Table 1:** Characteristics of soil microbial communities under different treatments.

Microbial community	CTNS	CTS	NTNS	NTS	T	S	T × S
Total PLFAs (nmol g^−1^)	10.93 ± 0.05 d	24.74 ± 0.05 b	15.89 ± 0.06 c	36.14 ± 0.07 a	**	**	**
Bacterial PLFA (nmol g^−1^)	2.79 ± 0.05 d	8.40 ± 0.09 b	5.12 ± 0.05 c	14.40 ± 0.07 a	**	**	**
Gram-positive bacterial PLFA (nmol g^−1^)	2.00 ± 0.04 d	5.14 ± 0.09 b	3.37 ± 0.04 c	8.06 ± 0.03 a	**	**	**
Gram-negative bacterial PLFA (nmol g^−1^)	0.78 ± 0.02 d	3.26 ± 0.00 b	1.75 ± 0.01 c	6.33 ± 0.05 a	**	**	**
G+/G^−^	2.56 ± 0.04 d	1.58 ± 0.03 b	1.92 ± 0.01 c	1.27 ± 0.01 a	**	**	**
MUFA/STFA	0.04 ± 0.00 d	0.07 ± 0.00 b	0.05 ± 0.00 c	0.09 ± 0.00 a	*	**	*

Different letters denote significant differences among treatments. ^**^P < 0.01; ^*^P < 0.05; ns, not significant. CTNS, conventional intensive tillage with straw removal; CTS, conventional intensive tillage with straw returning; NTNS, no-tillage with straw removal; NTS, no-tillage with straw returning. T, tillage; S, straw; G^+^/G^−^, gram-positive bacteria/gram negative bacteria; MUFA/STFA, monounsaturated fatty acids/saturated fatty acids. Values are mean ± standard errors.

**Table 2:** Taxonomy of soil bacterial communities determined in the present study.

Phylum	Class	Order	Family	Genus	T	S	T*S
*Actinobacteria*	*Actinobacteria*	*Actinomycetales*	*Mycobacteriaceae*	*Mycobacterium*	ns	**	ns
*Bacteroidetes*	*Sphingobacteria*	*Sphingobacteriales*	*Cytophagaceae*	*Arcicella*	ns	**	ns
*Firmicutes*	*Bacilli*	*Bacillales*	*Bacillaceae*	*Bacillus*	**	**	ns
	*Clostridia*	*Clostridiales*	*Clostridiaceae*	*Clostridium*	ns	*	ns
*Gemmatimonadetes*	*Gemmatimonadetes*	*Gemmatimonadales*	*Gemmatimonadaceae*	*Gemmatimonas*	**	**	**
*Nitrospira*	*Nitrospira*	*Nitrospirales*	*Nitrospiraceae*	*Nitrospira*	ns	**	ns
*Planctomycetes*	*Planctomycetacia*	*Planctomycetales*	*Planctomycetaceae*	*Gemmata*	ns	**	**
		*Planctomycetales*	*Planctomycetaceae*	*Pirellula*	ns	**	**
		*Planctomycetales*	*Planctomycetaceae*	*Schlesneria*	ns	*	*
		*Planctomycetales*	*Planctomycetaceae*	*Zavarzinella*	ns	ns	ns
*Proteobacteria*	*Alphaproteobacteria*	*Caulobacterales*	*Caulobacteraceae*	*Caulobacter*	*	ns	*
		*Caulobacterales*	*Caulobacteraceae*	*Phenylobacterium*	ns	ns	ns
		*Rhizobiales*	*Hyphomicrobiaceae*	*Blastochloris*	*	**	*
		*Rhizobiales*	*Xanthobacteraceae*	*Pseudolabrys*	*	**	*
		*Rhodospirillales*	*Rhodospirillaceae*	*Telmatospirillum*	*	ns	ns
		*Sphingomonadales*	*Sphingomonadaceae*	*Sphingomonas*	*	*	ns
	*Betaproteobacteria*	*Burkholderiales*	*Burkholderiaceae*	*Burkholderia*	**	**	**
	*Gammaproteobacteria*	*Methylococcales*	*Methylococcaceae*	*Methylococcus*	ns	ns	ns
		*Pseudomonadales*	*Pseudomonadaceae*	*Pseudomonas*	**	**	**
		*Xanthomonadales*	*Xanthomonadaceae*	*Dokdonella*	**	**	**
		*Xanthomonadales*	*Xanthomonadaceae*	*Dyella*	ns	ns	ns
		*Xanthomonadales*	*Xanthomonadaceae*	*Rhodanobacter*	ns	**	ns
		*Xanthomonadales*	*Xanthomonadaceae*	*Rudaea*	*	**	*

Different letters denote significant differences among treatments. ^**^P < 0.01; ^*^P < 0.05; ns, not significant. T, tillage; S, straw; Values are mean ± standard errors.

**Table 3:** Relationships between SOC and bacterial genera based on stepwise regression analysis.

Constant and dependent variables	Coefficient of regression estimate	P value	Significance
Intercept	15.284	**	R^2^ = 0.99
*Gemmatimonas*	0.001	*	
*Rudaea*	0.004	**	
*Sphingomonas*	0.009	*	
*Pseudomonas*	0.089	**	
*Dyella*	0.069	**	
*Burkholderia*	−0.093	**	
*Clostridium*	0.009	*	
*Pseudolabrys*	−0.018	**	
*Arcicella*	−0.015	*	
*Bacillus*	0.111	**	

^*^P < 0.05; ^**^P < 0.01.

## References

[b1] Mathew R. P., Feng Y., Githinji L., Ankumah R. & Balkcom K. S. Impact of No-tillage and conventional tillage on soil microbial communities. Appl. Environ Soil Sci. 10.1155/2012/548620 (2012).

[b2] Guo L. J., Lin S., Liu T. Q., Cao C. G. & Li C. F. Effects of conservation tillage on topsoil microbial metabolic characteristics and organic carbon within aggregates under a rice (*Oryza sativa* L.) –wheat (*Triticum aestivum* L.) cropping system in central China. Plos One 11, e0146145 (2016). 26731654 10.1371/journal.pone.0146145 PMC4701456

[b3] Varvel G. E. & Wilhelm W. W. Long-term soil organic carbon as affected by tillage and cropping systems. Soil. Sci. Soc. Am. J. 74, 915–921 (2010).

[b4] Larkin R. P. Soil health paradigms and implications for disease management. Annu. Rev. Phytopathol. 53, 199–221 (2015). 26002292 10.1146/annurev-phyto-080614-120357

[b5] Sun R., Zhang X. X., Guo X., Wang D. & Chu H. Bacterial diversity in soils subjected to long-term chemical fertilization can be more stably maintained with the addition of livestock manure than wheat straw. Soil. Biol. Biochem. 88, 9–18 (2015).

[b6] Zhang S., Li Q., Lü Y., Zhang X. & Liang W. Contributions of soil biota to C sequestration varied with aggregate fractions under different tillage systems. Soil. Biol. Biochem. 62, 147–156 (2013).

[b7] Guo L. J., Zhang Z. S., Wang D. D., Li C. F. & Cao C. G. Effects of short-term conservation management practices on soil organic carbon fractions and microbial community composition under a rice-wheat rotation system. Biol. Fert. Soils 51, 65–75 (2015).

[b8] Murugan R., Koch H. J. & Joergensen R. G. Long-term influence of different tillage intensities on soil microbial biomass, residues and community structure at different depths. Biol. Fert. Soils 50, 487–498 (2014).

[b9] Wuest S. B., Caesar-TonThat T., Wright S. F. & Williams J. D. Organic matter addition, N, and residue burning effects on infiltration, biological, and physical properties of an intensively tilled silt-loam soil. Soil. Till. Res. 84, 154–167 (2005).

[b10] Goyal S. & Sindhu S. S. Composting of rice straw using different inocula and analysis of compost quality. Microbiol. J. 1, 126–138 (2011).

[b11] Mau R. L. . Linking soil bacterial biodiverstity and soil carbon stability. ISME J. 9, 1477–1480 (2015). 25350158 10.1038/ismej.2014.205 PMC4438316

[b12] Yue H. W. . The microbe-mediated mechanisms affecting topsoil carbon stock in Tibetan grasslands. Int. Soc. Microb. Ecol. 9, 2012–2020 (2015). 10.1038/ismej.2015.19 PMC4542033 25689025

[b13] Acosta-Martínez V. . Microbial community composition as affected by dryland cropping systems and tillage in a Semiarid Sandy soil. Diversity 2, 910–931 (2010).

[b14] Figuerola E. L. . Bacterial indicator of agricultural management for soil under no-till crop production. Plos One 7, e51075 (2012). 23226466 10.1371/journal.pone.0051075 PMC3511350

[b15] Neumann D., Heuer A., Hemkemeyer M., Martens R. & Tebbe C. C. Importance of soil organic matter for the diversity of microorganisms involved in the degradation of organic pollutants. ISME J. 8, 1289–1300 (2014). 24430482 10.1038/ismej.2013.233 PMC4030228

[b16] Zhao J., Wang B. Z. & Jia Z. J. U. Phylogenetically distinct phylotypes modulate nitrification in a paddy soil. Appl. Environ. Microbiol. 81, 3218–3227 (2015). 25724959 10.1128/AEM.00426-15 PMC4393434

[b17] Ekblad A. . The production and turnover of extramatrical mycelium of ectomycorrhizal fungi in forest soils: role in carbon cycling. Plant Soil 366, 1–27 (2013).

[b18] Clemmensen K. E. . Roots and associated fungi drive long-term carbon sequestration in boreal forest. Science 339, 1615–8 (2013). 23539604 10.1126/science.1231923

[b19] Witt. C. . Crop rotation and residue management effects on carbon sequestration, nitrogen cycling and productivity of irrigated rice systems. Plant Soil. 225, 263–278 (2012).

[b20] Govaerts B. . Infiltration, soil moisture, root rot and nematode populations after 12 years of different tillage, residue and crop rotation managements. Soil. Till. Res. 94, 209–219 (2007).

[b21] Grosbellet C., Vidal-Beaudet L., Caubel V. & Chapentier S. Improvement of soil structure formation by degradation of coarse organic matter. Geoderma 162, 27–38 (2011).

[b22] Zhang B., Li Y., Ren T. & Tian C. J. Short-term effect of tillage and crop rotation on microbial community structure and enzyme activities of a clay loam soil. Biol. Fert. Soils 50, 1077–1085 (2014).

[b23] Zhu L., Hu N., Yang M. F., Zhan X. & Zhang Z. Effects of different tillage and straw return on soil organic carbon in a rice-wheat rotation system. Plos One 9, e88900 (2014). 24586434 10.1371/journal.pone.0088900 PMC3930598

[b24] Kumari M. . Soil aggregation and associated organic carbon fractions as affected by tillage in a rice-wheat rotation in North India. Soil Sci. Soc. Am. J. 75, 560–567 (2011).

[b25] Guo J. H. . Significant acidification in major Chinese croplands. Science 327, 1008–1010 (2010). 20150447 10.1126/science.1182570

[b26] Ding L. L., Cheng H., Liu Z. F. & Ren W. W. Experimental warming on the rice-wheat rotation agro-ecosystem. Plant. Sci. J. 31, 49–56 (2013).

[b27] Editorial Board of China Agriculture Yearbook China. Agriculture Yearbook 2009, Electronic Edition. China Agriculture Press, Beijing, China (2012).

[b28] Wolfarth F., Schrader S., Oldenburg E. & Weinet J. Nematode-collembolan-interaction promotes the degradation of Fusarium biomass and deoxynivalenol according to soil texture. Soil. Biol. Biochem. 57, 903–910 (2013).

[b29] Swedrzyńska D., Małecka I., Blecharczyk A., Swedrzyński A. & Starzyk J. Effects of various long-term tillage systems on some chemical and biological properties of soil. Pol. J. Environ. Studies 22, 1835–1844 (2013).

[b30] Wang Y. . Long-term impact of farming practices on soil organic carbon and nitrogen pools and microbial biomass and activity. Soil. Till. Res. 117, 8–16 (2011).

[b31] Hammesfahr U., Heuer H., Manzke B., Smalla K. & Thiele-Bruhn S. Impact of the antibiotic sulfadiazine and pig manure on the microbial community structure in agricultural soils. Soil. Biol. Biochem. 40, 1583–1591 (2008).

[b32] Bossio D. A. & Scow K. M. Impacts of carbon and flooding on soil microbial communities: phospholipid fatty acid profiles and substrate utilization patterns. Microb. Ecol. 35, 265–278 (1998). 9569284 10.1007/s002489900082

[b33] Wang J. J. . Effects of tillage and residue management on soil microbial communities in north china. Plant. Soil. Environ. 58, 28–33 (2012).

[b34] Eynard A., Schumacher M. J., Lindstrom M. J., Malo D. D. & Kohl R. A. Effects of aggregate structure and organic C on wettability of Ustolls. Soil. Till. Res. 88, 205–216 (2006).

[b35] Zeytin S. & Baran A. Influences of composted hazelnut husk on some physical properties of soils. Bioresource Technol. 88, 241–244 (2003). 10.1016/s0960-8524(03)00005-1 12618046

[b36] Koeck D. E., Pechtl A., Zverlov V. V. & Schwarz W. H. Genomics of cellulolytic bacteria. Curr. Opin. Biotech. 29, 171–183 (2014). 25104562 10.1016/j.copbio.2014.07.002

[b37] Monserrate E., Leschine S. B. & Canale-Parola E. *Clostridium hungatei* sp. nov., a mesophilic, N_2_-fixing cellulolytic bacterium isolated from soil. Int. J. Syst. Evol. Microbiol. 51, 123–132 (2001). 11211249 10.1099/00207713-51-1-123

[b38] Suresh K., Prakash D., Rastogi N. & Jain R. K. *Clostridium nitrophenolicum* sp. Nov., a novel anaerobic p-nitrophenol-degrading bacterium, isolated from a subsurface soil sample. Int. J. Syst. Evol. Microbiol. 57, 1886–1890 (2007). 17684276 10.1099/ijs.0.64604-0

[b39] Desvaux M., Guedon E. & Petitdemange H. Cellulose catabolism by *Clostridium cellulolyticum* growing in batch culture on defined medium. Appl. Environ. Microbiol. 66, 2461–2470 (2000). 10831425 10.1128/aem.66.6.2461-2470.2000 PMC110559

[b40] Koukiekolo R. . Degradation of corn fiber by *Clostridium cellulovorans* cellulases and hemicellulases and contribution of scaffolding protein CbpA. Appl. Environ. Microbiol. 71, 3504–3511 (2005). 16000754 10.1128/AEM.71.7.3504-3511.2005 PMC1168997

[b41] Takaichi S., Maoka M., Takasaki K. & Hanada S. Carotenoids of *Gemmatimonas aurantiaca* (*Gemmatimonadetes*): identification of a novel carotenoid, deoxyoscillol 2-rhamnoside, and proposed biosynthetic pathway of oscillol 2, 29-dirhamnoside. Microbiology 156, 757–763 (2010). 19959572 10.1099/mic.0.034249-0

[b42] Glissmann K. & Conrad R. Fermentation pattern of methanogenic degradation of rice straw in anoxic paddy soil. FEMS Microb. Ecol. 31, 117–126 (2000). 10.1111/j.1574-6941.2000.tb00677.x 10640665

[b43] Glissmann K. & Conrad R. Saccharolytic activity and its role as a limiting step in methane formation during the anaerobic degradation of rice straw in rice paddy soil. Biol. Fert. Soils 35, 62–67 (2002).

[b44] Glissmann K., Weber S. & Conrad R. Localization of processes involved in methanogenic in degradation of rice straw in anoxic paddy soil. Environ. Microbiol. 3, 502–511 (2001). 11578311 10.1046/j.1462-2920.2001.00212.x

[b45] Yamada T. . *Bellilinea caldifistulae* gen. nov., sp. nov. and *Longilinea arvoryzae* gen. nov., sp. nov., strictly anaerobic, filamentous bacteria of the phylum *Chloroflexi* isolated from methanogenic propionate-degrading consortia. Int. J. Syst. Evol. Microbiol. 57, 2299–2306 (2007). 17911301 10.1099/ijs.0.65098-0

[b46] Takeuchi M., Hamana K. & Hiraishi A. Proposal of the genus *Sphingomonas sensu stricto* and three new genera, *Sphingobium, Novosphingobium* and *Sphingopyxis*, on the basis of phylogenetic and chemotaxonomic analyses. Int. J. Syst. Evol. Microbiol. 51, 1405–1417 (2001). 11491340 10.1099/00207713-51-4-1405

[b47] Peng J., Lü Z., Rui J. & Lu Y. Dynamics of the methanogenic archaeal community during plant residue decomposition in an anoxic rice field soil. Appl. Environ. Microbiol. 74, 2894–2901 (2008). 18344350 10.1128/AEM.00070-08 PMC2394899

[b48] Ayres E., Steltzer H., Berg S. & Wall D. H. Soil biota accelerate decomposition in high-elevation forests by specializing in the breakdown of litter produced by the plant species above them. J. Ecol. 97, 901–912 (2009).

[b49] Yin C., Jones K. L., Peterson D. E., Garrett K. A. & Hulbert S. H. Members of soil bacterial communities sensitive to tillage and crop rotation. Soil. Biol. Biochem. 42, 2111–2118 (2010).

[b50] Blair G. J., Lefory R. D. B. & Lise L. Soil carbon fractions based on their degree of oxidation and the development of a carbon management index for agricultural system. Aust. J. Agric. Res. 46, 1459–1466 (1995).

[b51] Peiffer J. A. . Diversity and heritability of the maize rhizosphere microbiome under field conditions. PNAS 110, 6548–6553 (2013). 23576752 10.1073/pnas.1302837110 PMC3631645

[b52] Zhao J. C. . Impact of enhanced *staphylococcus* DNA extraction on microbial community measures in cystic fibrosis sputum. Plos One 7, e33127 (2012). 22412992 10.1371/journal.pone.0033127 PMC3297625

[b53] Fierer N., Hamady M., Lauber C. L. & Jackson R. B. The influence of sex, handedness, and washing on the diversity of hand surface bacteria. PNAS 105, 17994–17999 (2008). 19004758 10.1073/pnas.0807920105 PMC2584711

[b54] Caporaso J. G. . QIIME allows integration and analysis of high-throughput community sequencing data. Nat. Methods 7, 335–336 (2010). 20383131 10.1038/nmeth.f.303 PMC3156573

[b55] Edgar R. C., Haas B. J., Clemente J. C., Quince C. & Knight R. Uchime improves sensitivity and speed of chimera detection. Bioinformatics 27, 2194–2200 (2011). 21700674 10.1093/bioinformatics/btr381 PMC3150044

[b56] Groemping U. Relaimpo: relative importance of regressors in linear models. *R package version 2.2-2* http://cran.r-project.org/web/packages/relaimpo/index.html (2013).

[b57] Grace J. B., Anderson T. M., Smith M. D., Seabloom E. & Andelman S. J. Does species diversity limit productivity in natural grassland communities? Ecol. Letters 10, 680–689 (2007). 10.1111/j.1461-0248.2007.01058.x 17594423

